# Horn possession reduces maneuverability in the horn-polyphenic beetle, Onthophagus nigriventris

**DOI:** 10.1673/2006_06_21.1

**Published:** 2006-09-22

**Authors:** Richard Madewell, Armin P. Moczek

**Affiliations:** Department of Biology, Indiana University, Bloomington IN

**Keywords:** alternative reproductive tactics, horned beetle, phenotypic plasticity, polyphenism, sexual selection

## Abstract

Alternative male morphologies are common in a wide range of organisms and particularly extreme in horned beetles. Here, large males (majors) commonly develop extravagant weaponry such as horns or enlarged mandibles, whereas small males (minors) develop only rudimentary traits. In some taxa, including the genus Onthophagus, the transition from minors to majors occurs over a very small range of body sizes causing intermediate morphologies to be rare or absent from natural populations. Several studies have shown that majors use horns as weapons during male combat over females and that the possession of horns increases male fighting success, and presumably fitness. However, the advantages of a hornless morphology, if any, have remained elusive. Here the alternative male morphs are examined in the horn-polyphenic beetle Onthophagus nigriventris. In particular, the hypothesis was tested that lack of horns in minors increases their maneuverability inside tunnel systems in which these males sneak matings from major males. Using a simple behavioral assay the effects of horn possession on maneuverability were quantified inside an artificial tunnel. Minors were found to be significantly more mobile compared to majors. No such differences were found in mobility between similarly small and large females, which always lack horns. This suggests that mobility differences observed among male morphs are due to the presence or absence of horns rather than differences in body size. This notion was further supported in a second experiment in which surgical removal of horns significantly improved maneuverability, while subsequent re-attachment of horns reversed this effect. These results suggest that lack of horns increases male maneuverability inside tunnels and may thus be advantageous in the context of the particular social niche inhabited by minor males. The results are discussed in the context of the evolutionary ecology of horn-polyphenic beetles.

## Introduction

Alternative male phenotypes are widespread in species with intense sexual selection, and often involve the expression of alternative aggressive fighter and non-aggressive sneaker morphs among competing males (Andersson 1989; West-Eberhard 2003). In most cases studied thus far fighter-sneaker dimorphisms are closely tied to male body size, with physically larger males engaging in fighting behavior to acquire mating opportunities, whereas smaller males engage in non-aggressive sneaking behaviors (Moczek and Emlen 2000). In many taxa such size-dependent expression of reproductive behaviors is also thought to have facilitated the evolution of corresponding alternative morphologies, such as exaggerated weaponry in fighter but not sneaker morphs (Shuster and Wade 2003). Discontinuous, size-dependent expression of male secondary sexual traits is particularly conspicuous in many beetle taxa, including the greatly exaggerated horns of many scarab beetles (Arrow 1951). Species in the genus Onthophagus often exhibit particularly extreme size-dependent expression of male horns, largely determined by differences in quantity and quality of food provisioned for larvae by their mothers in the form of brood balls. Typically, only males with access to optimal feeding conditions eclose at a large body size and express fully developed horns, whereas males with access to suboptimal feeding conditions eclose at a smaller body size and remain largely, or entirely, hornless (Emlen 1994; Hunt and Simmons 1997; Moczek and Emlen 1999). The transition from largely hornless (minor) morphs to fully horned (major) morphs often occurs over a surprisingly narrow body size range. As a consequence of this threshold action natural populations often exhibit a bimodal distribution of horn lengths, and intermediate phenotypes are typically rare. Several studies have now shown that beetle horns are used primarily as weapons in male-male combat (Eberhard 1978, 1979, 1982; Otronen 1988; Rassmussen 1994) and that the possession of long horns measurably improves a male's chances of winning fights against other males (Emlen 1997; Moczek and Emlen 2000). Thus, large males that engage in fighting behavior clearly benefit from the expression of large horns. However, the absence of horns in small males and the paucity of intermediate morphologies in natural populations are far less well understood, and only two studies have provided some insight into the possible selective significance of hornlessness (Moczek and Emlen 2000; Hunt and Simmons 2001).

Several hypotheses have been proposed to explain why smaller males exhibit greatly reduced horn expression, and why the transition from minor to major males often occurs over an extremely short range of body sizes, causing intermediate morphologies to be rare in nature. Hunt and Simmons (1997) observed a positive correlation between extent of horn expression, length of larval development and larval mortality in the horn polyphenic beetle Onthophagus taurus. This result suggested that by remaining hornless small males may be able to avoid these costs, however, a subsequent more detailed study (Moczek and Nijhout 2002) failed to replicate Hunt and Simmon's (1997) original correlation. Alternatively, Nijhout and Emlen (1999; see also Emlen 2001; Moczek and Nijhout 2004) showed that growth of horns appears to trade-off with the growth of other structures during larval development such as eyes, antennae, wings or genitalia. This suggested that individuals that develop disproportionately large horns may be constrained to develop disproportionately smaller, and possibly less functional, versions of other traits, and that smaller males may be able to avoid such costs by remaining hornless. Both hypotheses help explain why small males may gain a selective advantage by expressing relatively smaller horns, but fail to explain the sudden transition from largely hornless to fully horned male shapes observed in many species. A third hypothesis, originally put forward by Emlen (Emlen 1997; Moczek and Emlen 2000), addressed this issue by suggesting that hornlessness may be a direct adaptation to the social niche inhabited by small males. Small male Onthophagus typically rely on a high degree of agility inside a complex tunnel system underneath dung pads to locate and mate with females in the presence of horned guarding males. The possession of horns may reduce male maneuverability inside tunnels and thus be directly detrimental to the performance of males that engage in sneaking behaviors. If correct this would suggest that fighting and sneaking behaviors generate a disruptive selection environment, favoring long horns in males large enough to profitably engage in fighting behavior, but lack of horns in smaller males. In turn this would also help explain the sudden transition from horned to hornless morphs observed in natural populations, as males with intermediate morphologies would be expected to be inferior fighters and sneakers and thus selected against in both contexts (Emlen and Nijhout 2000). However, although intuitively appealing, evidence in favor of a mobility-handicap due to horn possession is largely anecdotal and only a single study has been able to provide some supporting behavioral data on one species of horn-dimorphic beetle (Moczek and Emlen 2000). Furthermore, a subsequent study on the same species (Hunt and Simmons 2001) was unable to detect a significant negative effect of horn length on the fitness of minor males, contrary to what would be expected if disruptive selection was operating on male morphology. Thus, the available evidence to characterize the selective conditions that shape male morphological diversity in horned beetles remains largely lacking and further studies on a wider range of species are clearly needed.

Here we focus on a previously unstudied species of horn-dimorphic beetle, Onthophagus nigriventris, which expresses one of the more extreme male dimorphisms of the genus. A straightforward behavioral approach is used in combination with phenotypic manipulations to test experimentally whether horn possession measurably affects male maneuverability in this species.

**Figure 1:**
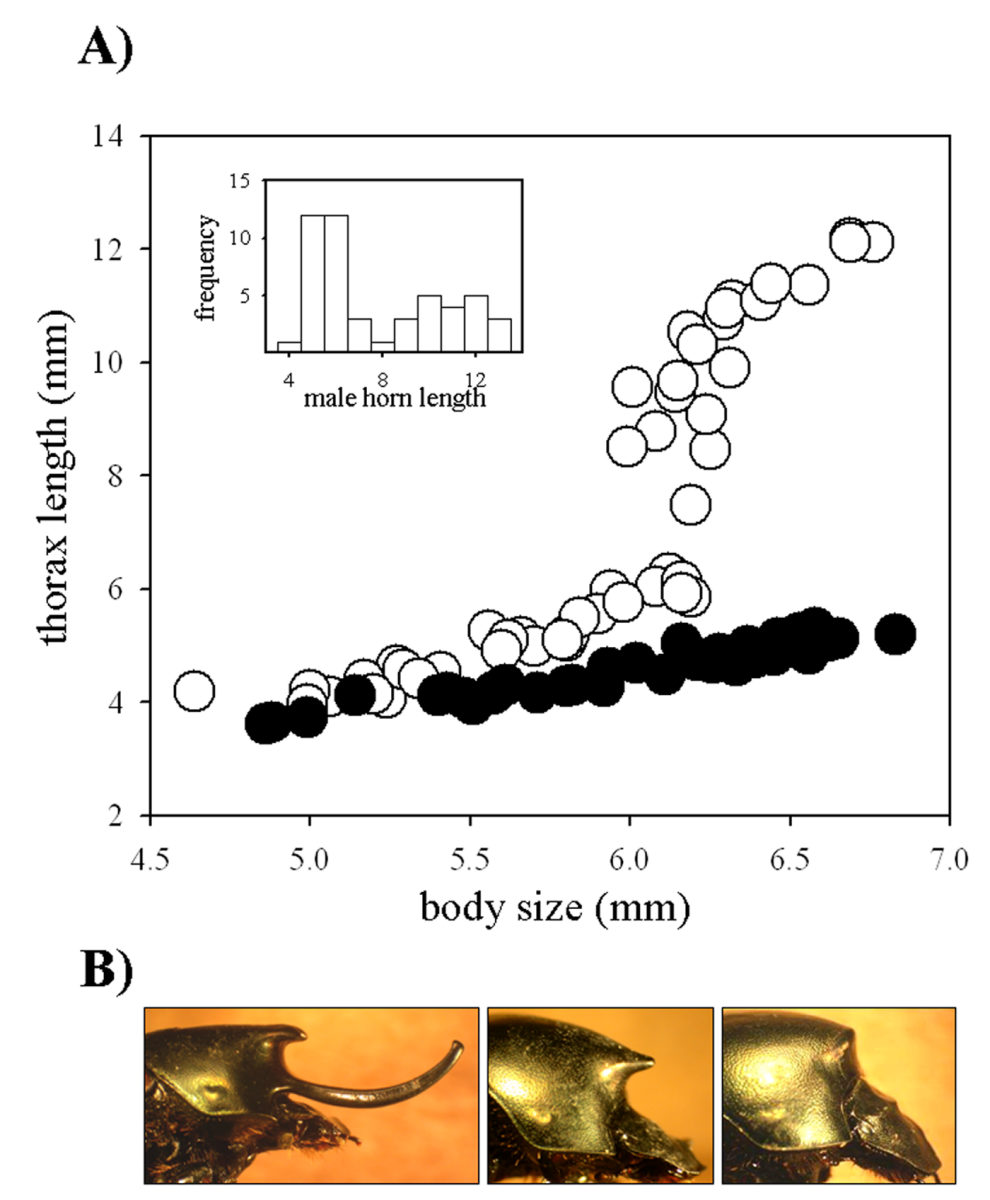
Phenotypic variation in Onthophagus nigriventris. **A:** Scaling relationship between body size (x-axis) and horn length (y-axis) of male (open circles) and female (solid circles) O. nigriventris. Body size was measured as pronotum width (see Moczek and Emlen 1999 for justification). Horn length was measured as pronotum length. **B:** Typical male and female phenotypes. Left: large, horned (major) male. Center: small, hornless (minor) male. Right: female.

Large male Onthophagus nigriventris express a single, long and curved, medial pronotal horn, produced during a period of explosive growth during the prepupal stage during late larval development (reviewed in Moczek 2006). This large prothoracic horn is reduced to a short and pointy rudiment in small males and absent in all females. In addition, large male adults also express a small, more posterior thoracic outgrowth in a location similar to the horn rudiment of small males. However, unlike the long horn in large males and horn rudiment in small males, this second, more posterior outgrowth of large males arises developmentally from sculpting and retraction of pupal horn tissue around the area of the final outgrowth rather than active growth. Similar sculpting and retraction of pupal tissue do not take place in small males and females (Moczek 2006). Males exhibit great discontinuity in the scaling relationship between body size and horn length (Fig. 1) and the transition from rudimentary to complete horn expression occurs over a narrow body size range. The sigmoid nature of the scaling relationship is typical for beetles in this genus, and effectively divides males into two relatively discrete morphs. Intermediate morphologies exist but are relatively rare (Fig. 1).

 O. nigriventris breeds, develops, and behaves similar to other onthophagine species studied previously (Cook 1990; Emlen 1997; Moczek and Emlen 2000;Moczek et al. 2002). Female O. nigriventris reproduce by provisioning dung in the form of brood balls in subterranean tunnels. Brood balls contain a single egg and represent the sole amount of food available to developing larvae, which complete larval and pupal development inside. Male O. nigriventris compete for access to tunnels and females.

## Material and Methods

Onthophagus nigriventris is a dung beetle native to the highlands of Kenya, but exotic populations exist in Australia and Hawaii. Animals used in the present study were part of a laboratory colony derived from a population on Manoa, Hawaii, and maintained in an insectary at Indiana University at a 16h:8h light: dark cycle and *ad libitum* food conditions.

### Maneuverability assay

Maneuverability was quantified by allowing beetles to run through and turn around inside an artificial tunnel consisting of a clear plastic tube with a 13 mm interior diameter. Turning around inside tunnels is a task performed by males and females on a regular basis, as observed during pilot observations of O. nigriventris using ant farms. (Moczek unpublished, for similar observations in other species see Emlen 1997, Moczek and Emlen 1999). Tunnels dug by O. nigriventris vary in diameter from at least 6mm (dug by small individuals) to approximately 18mm at tunnel intersections. Using trial runs at a variety of tunnel diameters we determined 13mm as the tunnel diameter that allowed >95% of all males to eventually complete a turn-around inside the tunnel. At the beginning of the assay we placed a beetle head first into one end of the tunnel while a bright light stimulus was presented at the other end. Beetles responded reliably to the light stimulus by walking toward the light. Light orientation was then reversed, which invariably caused beetles to attempt to turn around inside the tunnel. We quantified maneuverability by measuring the time it took a beetle to completely turn around inside its artificial tunnel. Turn-around performances were recorded to the nearest 0.1 second using a handheld stopwatch.

### Experiment 1

To examine the effects of horn possession on maneuverability correlated changes in body size had to be control for. To do so female O. nigriventris were used as controls. Females exhibit the same range of body sizes as males yet are always hornless. We used handheld digital calipers to measure thorax widths (as an estimate of body size; for justification see Moczek and Emlen 1999) of male and female O. nigriventris and then divided them into four different categories: (i) large, horned males (6.2–7.0mm thorax width); (ii) small, hornless males (5.2–6.0mm); (iii) large females (6.2–7.0mm); and (iv) small females (5.2–6.0mm). Maneuverability of 20 individuals in each category was quantified using the assay described above. Each individual was only used once. If horn possession alone affects maneuverability reduced performance was predicted in major males but not in minors, large females and small females. Alternatively, if body size affects maneuverability we predicted reduced performance in major males as well as large females.

### Experiment 2

In this experiment horn possession was experimentally manipulated in individual majors. Ten large, horned males were examined using the same assay as outlined above through three consecutive trials: i) with their horn intact, ii) with their horn removed, iii) with their horn re-attached. Each individual was tested three times within each experimental trial and an average was used for further analysis. We removed horns at their base using micro-scissors immediately following the first trial. Males were then given 60 minutes to recover. Horns were re-attached using CrazyGlue™ immediately following the second trial, and males were given at least 30 minutes to recover. Surgery did not appear to injure males. The medial horn is largely solid, contains neither muscles, nerves, nor hemolymph, and little to no bleeding occurred following surgery. Animals were obtained from a colony fed *ad libitum* at the time of the experiment, and great care was taken to keep beetles in a moist environment throughout the entire experiment to minimize any effects of dehydration on performance. At the end of the experiment beetles were released back into the colony and remained alive for at least several weeks following the experiment, as observed through weekly clean-up of dead animals. If horn possession impedes male maneuverability elevated performance (i.e. shorter turn-around times) was predicted in males whose horns had been removed, and a reversal of this effect after horns were re-attached.

### Statistical analysis

Two-tailed t-tests were used to compare performances in experiment 1. Results are presented as *p*_dgf , critical T_ = test statistic. To analyze results from experiment 2 Wilcoxon paired-signed rank tests were used to test for differences in individual performance after horn removal and re-attachment, respectively. Results are presented as *p*_W+/W−_ = test statistic.

## Results

**Figure 2:**
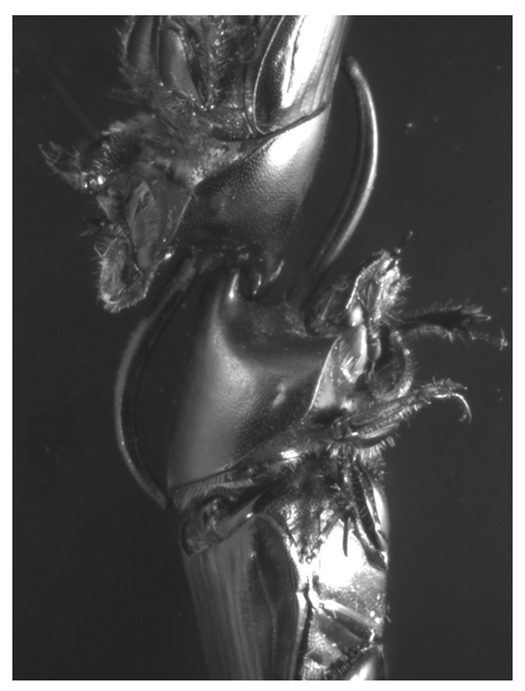
Fighting position of horned male O. nigriventris as observed in ant farms, illustrated using dead specimens. Shown are head and thorax of two males interlocked in typical fighting position (see text for further details).

Pilot observations using ant farms similar to those used by Moczek and Emlen (2000) indicated that in order to access breeding females, O. nigriventris males use a behavioral repertoire largely similar to that of other Onthophagus species (Moczek, unpublished). In each of eight fights staged between two fully horned males, competitors assumed a characteristic fighting position (Fig. 2). Opponents attacked each other head on, but the dorsoventral orientation of opponents within tunnels was opposite to one another (Fig 2). By attacking each other head on yet with backs oriented in opposite directions, male were able to interlock with their horns in a peculiar fashion. During fights, the long medial anterior horn of each male smoothly fit around the prothorax of his opponent. In some cases the tip of the horn is inserted into the space between thorax and abdomen, while the short, more posterior horn and associated hollowing in the cuticle anterior to it served as a receptacle of the short posterior horn of the opponent. Interlocked in this fashion, beetles engaged in shoving contests lasting 9.3 (±3.1) minutes (n = 8; for an excellent, detailed examination of a similar morphological situation see Eberhard and Garcia-C. 2000, Eberhard et al. 2000). Males entering the tunnel were either expelled by the tunnel owner or managed to maneuver their opponent to a location in the tunnel where they could pass him, turn around, and expel the former owner themselves. In all 8 fights observed winners remained with the female for at least the next 24 hours of brood ball production. In none of these cases did losers attempt to re-enter the tunnel for at least 2 hours following their initial defeat. Hornless, minor males, on the other hand, quickly withdrew from fights in each trial (mean fight duration 1.5 (± 0.34) minutes; n = 6 staged fights between one horned and one hornless males) yet in each case remained within the vicinity of the tunnel entrance and repeatedly attempted to re-enter the tunnel, however without success in any of the 6 trials observed. An obvious use for the rudimentary horn in small males was not determined.

Combined, these pilot observations suggested that apart from a species-specific fighting position, male O. nigriventris seem to rely on a qualitatively very similar behavioral repertoire as has already been documented in great detail for other onthophagine species (O. binodis:Cook 1990; O. acuminatus: Emlen 1997; O. taurus:Moczek and Emlen 2000;Moczek et al. 2002). We therefore focused our experiments to address a particular question largely unexplored by previous studies: does horn possession reduce maneuverability in horn dimorphic beetles, or inversely, do hornless males experience increase maneuverability by not investing in the development of large horns.

### Experiment 1

**Figure 3:**
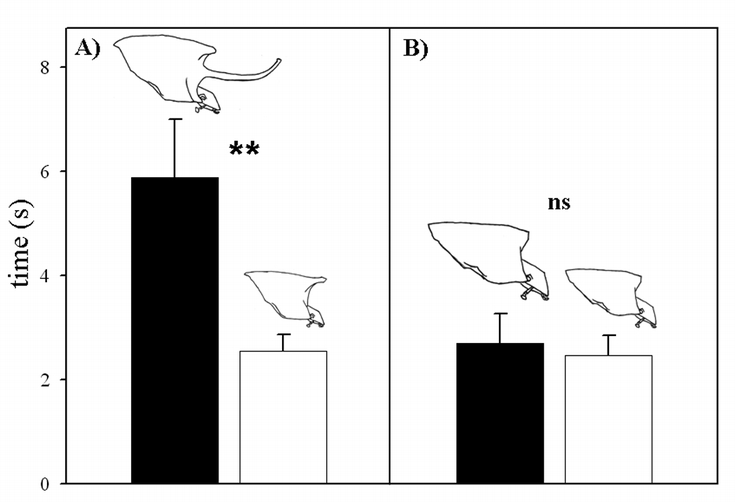
Mobility as a function of body size and horn length in O. nigriventris. Shown are mean mobility performances of (A) large, horned males and small, hornless males and (B) large and small females.

Large, horned males required significantly more time to completely turn around inside artificial tunnels compared to their small, hornless counterparts (*p*_48, 2.01_ = 0.006; Fig. 3). Large and small females, however, performed equally well (*p*_48, 2.01_ = 0.74) and similar to small males (Fig. 3). These results suggest that differences in performance between male morphs cannot be attributed to differences in body size, but instead appear to be due to the presence or absence of a horn.

### Experiment 2

**Figure 4:**
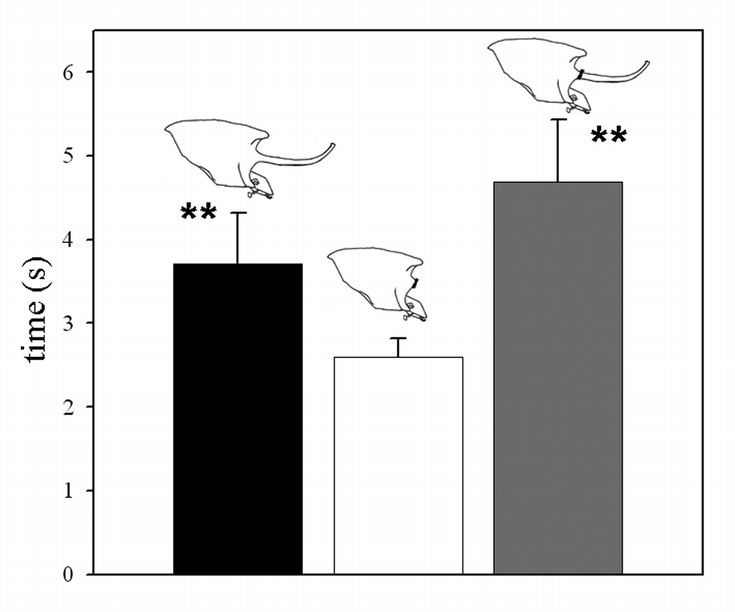
Mobility as a function of horn possession in large, horned O. nigriventris. Shown are mean mobility performances of large males with their horn intact, their horn removed, and their horn re-attached.

Individual majors with intact horns performed similar to the large, horned males in Experiment 1. Horn removal, however, significantly reduced turn-around times, and thus increased maneuverability (*p*_+54/−1_ = 0.039). This effect was reversed once horns were re-attached, resulting in a significant reduction of maneuverability back to the original level (*p*_+2/−53_ = 0.0059; Fig. 4). These results suggest that horns alone may be sufficient to impose a drastic reduction in male maneuverability inside tunnels.

## Discussion

The results of both experiments suggest that horn possession alone is sufficient to impose a possibly significant mobility handicap to horned males. In the first experiment small, hornless males consistently outperformed their large and horned counterparts. Interestingly, no difference was found in performance between small and large females, which instead both performed similar to small hornless males, which suggests that size itself may have a negligible effect on beetle mobility. In the second experiment surgical removal of horns similarly improved male performance, an effect that was reversed once horns were re-attached. The results thus support the hypothesis that the absence of large horns in small males improves male maneuverability inside tunnels. However, increased maneuverability may clearly not be the only advantage hornlessness can convey to a minor male. By not initiating horn growth small males may also be able to allocate resources to other structures whose normal function would either otherwise be compromised (such as eyes or antennae as suggested by Nijhout and Emlen 1999; Emlen 2001), or whose function would be improved beyond that of large horned males. A particularly interesting candidate structure are testes, whose sizes play an important role in determining a given males ability to increase his reproductive success via sperm competition (Simmons et al. 1999;Tomkins and Simmons 2000). While no data are available for O. nigriventris, studies on other onthophagine species have found that minor males have indeed developed significantly larger testes and ejaculate volumes compared to their major male counterparts (Simmons et al 1999;Tomkins and Simmons 2000). If correct, this would make the increase in maneuverability due to lack of horns reported here an *added* advantage to minor males.

Our study faces at least three possibly important limitations. First, the mobility assay relied on the use of an artificial, horizontal tunnel made of plastic tubing and light stimuli to manipulate beetle behavior. In nature, beetles run, compete, and mate inside subterranean tunnels dug through soil or sand (Cook 1990; Emlen 1997). Tunnels are of varying diameter and orientation, and may intercept with other tunnels (Moczek and Emlen 2000). Except near tunnel entrances beetles typically behave in complete darkness. Even though the diameter of the experimental tunnel was within the range of natural tunnel diameters, the assay thus relied on a relatively artificial environment to quantify male maneuverability. On the other hand, a critical advantage of the assay was that it could be standardized reliably across treatment groups. Performance-differences between treatment groups are therefore unlikely to be due to the assay, rather they are likely to reflect real differences in agility as a function of male horn phenotype. Surprisingly, relatively moderate sample sizes for each experiment were sufficient to detect measurable, and highly significant, mobility differences between horned and hornless male morphs, suggesting that horn possession has an immediate and drastic effect on male mobility inside tunnels.

Secondly, our results may only be applicable to a subset of horn-dimorphic beetles. O. nigriventris exhibits one of the more spectacular cases of male horn dimorphism, though many other Onthophagus species exhibit similarly extreme morphologies and intraspecific morphological variation (e.g. O. mahouti: Moczek 2005; O. watanabei: Moczek et al. 2004). While the magnitude of a mobility handicap is therefore clearly at least, in part, a function of the magnitude of horn development, we believe that the results can be extrapolated to other species within this genus. A second, related consideration concerns the importance of spatial context within which animals behave. A mobility advantage to small, hornless males may only exist, or be significant, in instances where individuals have to perform in confined spaces such as subterranean tunnels. This certainly applies to all species of the genus Onthophagus and many other dung beetle genera (e.g. Phanaeus, Caccobius) that also feature horned species, but it does not apply to may other taxa of horned beetles including the often spectacularly horned species in the subfamily Dynastinae (Siva-Jothy 1987; Kawano 1995, 2002; Mizunima 1999). Here, fights occur arboreally outside the confines of a tunnel, and it remains to be investigated whether fights over access to entrances to nesting sites or feeding sites may have the potential to impose their own spatial constraints that could possibly magnify mobility advantages to small, hornless, sneaking males.

The third and most conceptual challenge to our study lies in the fact that we were unable to measure fitness consequences of reduced or enhanced mobility. While the results suggest that the absence of horns increases maneuverability inside tunnels, this increase may have no effect on the reproductive success of minors and thus be selectively neutral. This is particularly noteworthy since a previous study also found behavioral evidence in support of a mobility handicap in horned O. taurus (Moczek and Emlen 2000), while a subsequent study on the same species failed to detect negative fitness effects of horn length on minor males (Hunt and Simmons 2001). It remains unclear at this point whether this absence of such negative fitness effects is indeed characteristic of onthophagine mating systems in general, confined to the particular species under study, or a limitation of the experimental design which quantified fitness as fertilization success over a 5 day period inside plastic buckets (Hunt and Simmons 2001).

On the other hand, results from several other studies suggest that the reproductive success of minor males, especially if it would be quantified over individual life time and under more natural conditions, is likely to be profoundly impeded by the possession of horns. Minor males rely on speed, agility, and reduced copulation duration to sneak matings from physically superior horned, major males (O. binodis: Cook 1990; O. acuminatus: Emlen 1997; O. taurus: Moczek and Emlen 2000). In O. taurus, for example, mating success appears to be directly related to a hornless male's ability to circumvent a guarding male during a sneaking attempt. If successful, horned males appear to be unable to sense the presence of a sneaker male and ignore extra-pair copulations. However, if hornless males do make contact with horned guarding males, e.g. by failing to retreat fast enough from a successful sneaking attempt, this is invariably followed by a mating between the guarding male and focal female (Moczek and Emlen 2000). This in turn is likely to severely detract from the sneaker male's fertilization success due to a generally high last-male fertilization advantage in onthophagine beetles (Hunt and Simmons 2000). The present study was able to detect a highly significant mobility difference as a function of horn-possession using single replicates and a moderate sample size. If these results are representative, the life time mobility advantage of hornless males is likely to be dramatic and, as a consequence, likely to positively affect the fitness of small, hornless males that do not rely on horns as weapons in male-male combat.

If correct this suggests that sneaking behavior in small males may indeed favor a hornless phenotype, opposite to the horned phenotype favored in males large enough to profitably engage in fighting behavior. In this scenario, beetles with a variety of horn sizes would be expected to be inferior fighters and sneakers because an intermediate horn size is not suitable for fighting with large, horned males yet at the same time would cause a significant handicap to a sneaking male, as shown here. Thus intermediates should be selected against in the context of either reproductive tactic. Combined, this would help explain the selective advantage of genotypes capable of facultative, size-dependent expression of hornless and horned male phenotypes and the often sudden, threshold-like transition between alternatives commonly observed in natural populations of onthophagine beetles. Clearly, direct estimates of male fitness as a function of body size and horn length in this and additional species will have to follow to further evaluate the significance of our results and of horns as mobility handicaps in horned beetles in general.
